# AMPA Receptor–mTOR Activation is Required for the Antidepressant-Like Effects of Sarcosine during the Forced Swim Test in Rats: Insertion of AMPA Receptor may Play a Role

**DOI:** 10.3389/fnbeh.2015.00162

**Published:** 2015-06-18

**Authors:** Kuang-Ti Chen, Mang-Hung Tsai, Ching-Hsiang Wu, Ming-Jia Jou, I-Hua Wei, Chih-Chia Huang

**Affiliations:** ^1^Institute of Basic Medical Science, China Medical University, Taichung, Taiwan; ^2^Department of Anatomy, China Medical University, Taichung, Taiwan; ^3^Department of Anatomy, College of Medicine, Taipei Medical University, Taipei, Taiwan; ^4^School of Chinese Medicine for Post Baccalaureate, I-Shou University, Kaohsiung, Taiwan; ^5^Department of Psychiatry, China Medical University Hospital, Taichung, Taiwan; ^6^Institute of Clinical Medical Science, China Medical University, Taichung, Taiwan; ^7^Department of Psychiatry, China Medical University, Taichung, Taiwan

**Keywords:** sarcosine, mTOR, NMDA, AMPA, depression

## Abstract

Sarcosine, an endogenous amino acid, is a competitive inhibitor of the type I glycine transporter and an *N*-methyl-d-aspartate receptor (NMDAR) coagonist. Recently, we found that sarcosine, an NMDAR enhancer, can improve depression-related behaviors in rodents and humans. This result differs from previous studies, which have reported antidepressant effects of NMDAR antagonists. The mechanisms underlying the therapeutic response of sarcosine remain unknown. This study examines the role of mammalian target of rapamycin (mTOR) signaling and α-amino-3-hydroxy-5-methylisoxazole-4-propionate receptor (AMPAR) activation, which are involved in the antidepressant-like effects of several glutamatergic system modulators. The effects of sarcosine in a forced swim test (FST) and the expression levels of phosphorylated mTOR signaling proteins were examined in the absence or presence of mTOR and AMPAR inhibitors. In addition, the influence of sarcosine on AMPAR trafficking was determined by analyzing the phosphorylation of AMPAR subunit GluR1 at the PKA site (often considered an indicator for GluR1 membrane insertion in neurons). A single injection of sarcosine exhibited antidepressant-like effects in rats in the FST and rapidly activated the mTOR signaling pathway, which were significantly blocked by mTOR inhibitor rapamycin or the AMPAR inhibitor 2,3-dihydroxy-6-nitro-7-sulfamoyl-benzo(f)quinoxaline (NBQX) pretreatment. Moreover, NBQX pretreatment eliminated the ability of sarcosine to stimulate the phosphorylated mTOR signaling proteins. Furthermore, GluR1 phosphorylation at its PKA site was significantly increased after an acute *in vivo* sarcosine treatment. The results demonstrated that sarcosine exerts antidepressant-like effects by enhancing AMPAR–mTOR signaling pathway activity and facilitating AMPAR membrane insertion.

**Highlights**
– A single injection of sarcosine rapidly exerted antidepressant-like effects with a concomitant increase in the activation of the mammalian target of rapamycin mTOR signaling pathway.
– The antidepressant-like effects of sarcosine occur through the activated AMPAR–mTOR signaling pathway.
– Sarcosine could enhance AMPAR membrane insertion via an AMPAR throughput.

## Introduction

Current antidepressants primarily comprise monoamine-targeting agents and have limited efficacy (Trivedi et al., 2006; Fekadu et al., 2009; Rizvi et al., 2014). Recently, modulation of the glutamatergic system has become an attractive strategy for discovering new-generation antidepressants (Shimizu-Sasamata et al., 1996; Skolnick, 1999; Krystal et al., 2002; Stewart and Reid, 2002; Hashimoto, 2011; Tokita et al., 2012). For example, ketamine, an *N*-methyl-d-aspartate receptor (NMDAR) antagonist, rapidly and sustainably ameliorates depressive symptoms in patients with major depression (Zarate et al., 2006b,c). Several preclinical reports have also indicated that NMDAR-antagonizing drugs, such as ketamine, MK-801, AP7, CGP37849, and CGP39551, have antidepressant-like properties in various animal models of depression (Kugaya and Sanacora, 2005; Maeng et al., 2008; Li et al., 2010). Sarcosine, an endogenous amino acid, is a competitive inhibitor of the type I glycine transporter (GlyT1) (Smith et al., 1992) and an NMDAR coagonist (Zhang et al., 2009). Because of these two properties, sarcosine can enhance NMDAR function. We previously conducted both rodent behavior tests and a trial of sarcosine treatment for major depression and demonstrated that sarcosine treatment elicits similar antidepressant-like effects in both acute and chronic stress models of depression and achieved a much higher remission rate than did a standard selective serotonin reuptake inhibitor (SSRI) treatment for major depression (Huang et al., 2013). Preclinical studies have also shown that the administration of other NMDAR enhancers, such as a reversible glycine transporter inhibitor, SSR504734, and an NMDAR coagonist, d-serine, has antidepressant and anxiolytic effects in depression and anxiety models (Depoortere et al., 2005; Malkesman et al., 2012). Studies have elucidated that the rapid activation of the mammalian target of rapamycin (mTOR) signaling pathway through stimulation of the α-amino-3-hydroxy-5-methyl-4-isoxazole propionic acid receptor (AMPAR) is a major mechanism of the NMDAR antagonist ketamine in exerting its rapid antidepressant effects (Maeng et al., 2008; Li et al., 2010). Similarly, studies have suggested that some glutamatergic system modulators, such as the metabotropic glutamate receptor mGluR_2/3_ antagonist LY341495, mGluR_5_ antagonist MTEP, and mGluR_7_ agonist AMN082, produce antidepressant-like effects through increased activation of mTOR, and some studies have shown that the effects of some of them are also dependent on AMPAR activation (Koike and Chaki, 2014; Palucha-Poniewiera et al., 2014). Until now, the molecular mechanisms underlying the antidepressant effects of sarcosine have been unclear. The present study was conducted to determine whether sarcosine increases the activation of mTOR signaling or AMPAR and whether the antidepressant-like effects of sarcosine require the stimulation of mTOR signaling or AMPAR.

Therefore, the effects of sarcosine in a forced swim test (FST) and the expression levels of phospho-mTOR (pmTOR), phosphor-extracellular signal-regulated protein kinase (pERK), and phospho-Akt (pAkt) in the hippocampus, an area directly implicated in mood regulation and antidepressant effectiveness (Nestler et al., 2002; Campbell and Macqueen, 2004) were examined in the absence or presence of an mTOR antagonist, rapamycin, and the selective AMPAR antagonist 2,3-dihydroxy-6-nitro-7-sulfamoyl-benzo(f)quinoxaline (NBQX). In addition, we assessed the possible involvement of AMPAR trafficking in the effects of sarcosine on mTOR activation by focusing on AMPAR membrane insertion, known to be induced by glycine (Lu et al., 2001) and involved in antidepressant effects (Du et al., 2007). The present study was specifically designed to obtain results, for the first time, of potential antidepressant-like effects of sarcosine on the mTOR signal pathway and AMPAR for understanding the molecular events caused by sarcosine.

## Materials and Methods

### Animals

Male Wistar rats, weighing 250–350 g, were used. Five rats were housed per cage, with food and water available *ad libitum* in the laboratory animal center, and maintained on a 12-h light–dark cycle (light, 07:00–19:00) at 23°C ± 1°C and in a 60% humidity-controlled environment. After at least a seven-day acclimation period in the center, the rats were transferred to the testing room and were immediately used for subsequent experiments. The study protocol was approved by the Institutional Animal Care and Use Committee of China Medical University, Taiwan.

### Study design

#### Experimental Protocols

**Figure 1:**
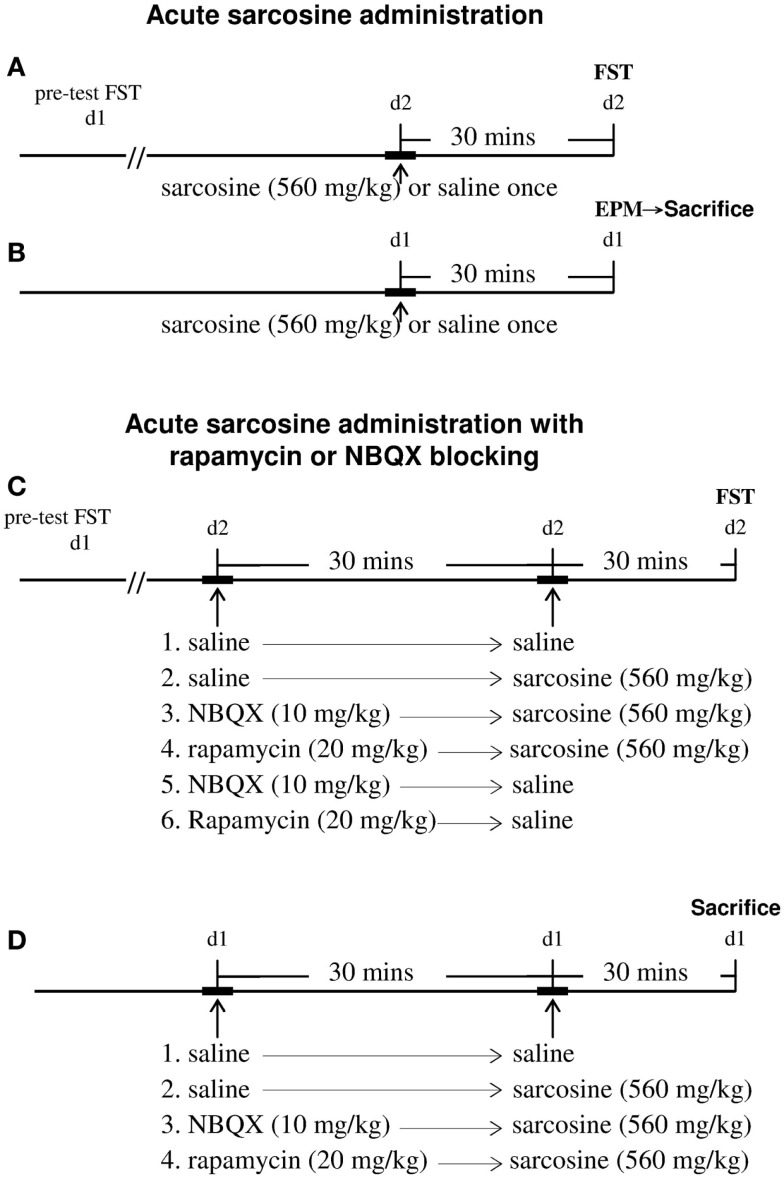
**Schemata demonstrating the timeline of the experiments for drugs administrations, behavioral tests, and time of sacrifice for western blots analysis**. For acute sarcosine administration **(A,B)**, rats were given saline or sarcosine (560 mg/kg, i.p.) once. The forced swim test (FST) was conducted 30 min later **(A)**. At 24 h before FST, rats had a 15-min conditioning swim. To evaluate the general locomotor activity, rats were administrated with saline or sarcosine (560 mg/kg, i.p.) once. The elevated plus-maze test (EPM) was conducted 30 min later **(B)**. Immediately after EPM, rats were sacrificed and then rapidly decapitated. The hippocampus was removed for biochemical analysis **(B)**. For acute sarcosine administration in the absence or presence of mTOR and AMPAR inhibitors **(C,D)**, either AMPA inhibitor (NBQX, 10 mg/kg, i.p.) or mTOR pathway inhibitor (rapamycin, 20 mg/kg, i.p.) was administrated 30 min before sarcosine (560 mg/kg, i.p.) or saline treatment. At 30 min after last injection, rats were then tested in an FST paradigm **(C)**. In a separate study **(D)**, naïve rats were randomly treated with either AMPA inhibitor (NBQX, 10 mg/kg, i.p.) or mTOR pathway inhibitor (rapamycin, 20 mg/kg, i.p.) was administrated 30 min before sarcosine (560 mg/kg, i.p.) treatment. Thirty minutes after last injection, rats were sacrificed and then rapidly decapitated. The hippocampus was removed for biochemical analysis.

Sarcosine (Merck Millipore, #807666), rapamycin (Toku-E, #R001), and NBQX (Tocris, #0373) were dissolved in saline and injected intraperitoneally (i.p.) in a volume of 0.01 mL/g of body weight. The naïve rats were randomly treated with saline (control) or sarcosine (560 mg/kg, i.p.) [as previously reported in our earlier study (Huang et al., 2013) to evoke antidepressant-like effects]. The FST was performed 30 min after treatment. In addition, rats first had a 15-min conditioning swim 24 h before the FST (Figure 1A). Each experimental group comprised 10 rats. To evaluate the general locomotor activity, in another experiment, naïve rats were treated with saline or sarcosine (560 mg/kg, i.p.), and the elevated plus-maze test (EPM) was conducted 30 min later (Figure 1B). Each experimental group comprised eight rats. Immediately after EPM, four rats in each group were sacrificed using an intramuscular injection of mixture of zoletil (30 mg/kg) and xylazine (10 mg/kg) followed by immediate decapitation. The hippocampus was removed and stored at −80°C for biochemical analysis.

In addition, the mTOR pathway inhibitor rapamycin or AMPAR inhibitor NBQX was used to determine whether sarcosine might induce antidepressant-like effects through these signaling pathways (Figure 1C). Saline, rapamycin (20 mg/kg, i.p.) (Cleary et al., 2008), or NBQX (10 mg/kg, i.p.) (Maeng et al., 2008) was administered 30 min before sarcosine (560 mg/kg, i.p.) or saline injection. Thirty minutes after the final injection, the rats were tested in an FST paradigm. Each experimental group comprised eight to nine rats. In a separate experiment, another 16 naïve rats were randomly divided into 4 groups, with 4 rats per group (Figure 1D). Saline, rapamycin (20 mg/kg, i.p.), or NBQX (10 mg/kg, i.p.) was administered 30 min before sarcosine (560 mg/kg, i.p.) injection. Thirty minutes after the final injection, the rats were sacrificed using an intramuscular injection of a mixture of zoletil (30 mg/kg) and xylazine (10 mg/kg), followed by immediate decapitation. The hippocampus was removed and stored at −80°C for biochemical analysis.

### Behavioral assays

#### Forced Swim Test

The FST was performed in an acrylic cylinder (diameter, 20 cm; height, 40 cm) filled to a height of 30 cm with 25°C water. Rats first had a 15-min conditioning swim before being placed in the swimming apparatus again 24 h later. Their behavior was monitored for 5 min after the administration of drugs or 0.9% saline (Porsolt et al., 1977; Cryan et al., 2005). The total periods of immobility during the 5-min testing period were recorded using the EthoVision Basic V 3.1 program (Noldus, Wageningen, Netherlands) and confirmed through direct observation. The effects of medication were evaluated using a computer and traditional manual methods. For computer scoring, immobility was set at 15% and a fixed averaging interval of 1 s was used for smoothing the mobility parameter. These settings were used to automatically acquire the activity of all rats in the FST. Manual scoring of the behavioral patterns was performed by an investigator who was blinded to the experimental conditions of the animals. We used a time-sampling technique whereby the predominant behavior in each 5-s period of the 300-s test was recorded (Detke et al., 1997). Climbing behaviors comprised upward movements of the forepaws along the side of the swim chamber. Swimming behavior was defined as movement (typically horizontal) throughout the swim chamber, which typically involved crossing into another quadrant. Immobility was assigned when no additional activity was observed other than that required to keep the rat’s head above water.

#### Elevated Plus-Maze Test

The EPM was placed in a dark room with a localized dim light (3 × 5 W) facing the apparatus and mounted 130 cm above the EPM’s surface. The rats were placed in the central square and their behaviors were monitored for 5 min after the administration of drugs or 0.9% saline. The numbers of open and closed arm entries and distance moved were recorded using the EthoVision Basic V 3.1 program. The total closed arm entries and distances moved were analyzed quantitatively as a relatively pure index of locomotor activity (Rodgers and Johnson, 1995; Hogg, 1996).

### Western blotting

Previously described methods for tissue processing and Western blotting were followed (Encinas et al., 2004). The dissected brain tissue was frozen in liquid nitrogen and then homogenized with 100 mL of lysis buffer by using a grinder on ice. Subsequently, 100 μg of solubilized proteins were separated using electrophoresis on a 10% polyacrylamide gel, transferred to nitrocellulose membranes, and stained with Ponceau Red for confirming equal protein loading. The membranes were blocked with 5% non-fat dry milk for 1 h, and then immunoreacted overnight at 4°C with rabbit polyclonal antiphospho-mTOR (Cell Signaling, #2971S) at a dilution of 1:1000, rabbit monoclonal anti-pERK (for detecting ERK1/2 MAPKs phosphorylated at Thr202/Tyr204 and Thr185/Tyr187, Millipore, #05-797R) at a dilution of 1:1000, rabbit polyclonal antiphospho-Akt1/PKBαser-473 (Millipore, #05-669) at a dilution of 1:1000, or rabbit polyclonal antiphospho-AMPA GluR1 Ser845 (Sigma, #A4477) at a dilution of 1:1000 primary antibodies. The nitrocellulose membranes were further processed for chemiluminescence detection (Santa Cruz) by using horseradish peroxidase-conjugated goat antirabbit secondary antibody diluted to 1000× (Santa Cruz) for 1 h at room temperature. Equal protein loading was confirmed by stripping the membranes, and then immunoreacting with β-actin (Millipore, #MAB1501) at a dilution of 1:2000, rabbit monoclonal anti-mTOR (Millipore, #04-385) at a dilution of 1:1000, Akt (Cell Signaling, #9272) at a dilution of 1:1000, p44/p42 ERK (Millipore, #05-1152) at a dilution of 1:1000, or rabbit polyclonal anti-AMPAR GluR1 (Millipore, #AB1504) at a dilution of 1:1000. Optical densities were quantified using a computer-assisted program (Gel-Pro Analyzer).

### Statistical analysis

Data of the animal studies were evaluated using a Student’s *t-*test or one-way analysis of variance, followed by a Tukey *post hoc* test, using SPSS 12.0 statistical software. All statistical tests were two-tailed, and results were considered significant at *p* < 0.05.

## Results

### Effects of a single injection of sarcosine

**Figure 2:**
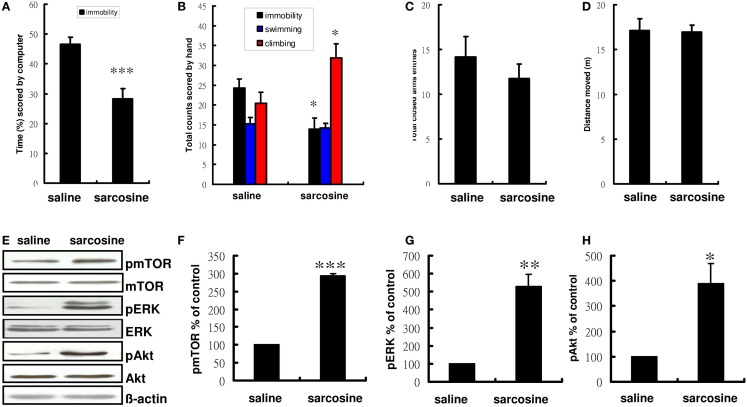
**The behaviors and representative Western blotting of rats in forced swim test after acute administration with saline or sarcosine (560 mg/kg, i.p.)**. In the acute treatment scored by computer **(A)**, the rats received a single injection of sarcosine prior to FST showed a significant reduction in percentage of immobility time. In the acute treatment scored manually **(B)**, the rats received a single injection of sarcosine prior to FST showed a considerable reduction in immobility and an increase in climbing. The general activity [**(C)**, numbers of closed arm entries and **(D)**, total distance moved] of rats in the elevated plus maze (EPM) after acute administration with saline or sarcosine. The total closed arm entries **(C)** and distance moved **(D)** in EPM were measured to determine if sarcosine could produce a general increase in general locomotor activity that could yield a false-positive result on the FST. At the doses tested, none increased locomotor activity. Western blots analysis shows a notably increased expression of pmTOR, pERK, and pAkt in rat hippocampus following acute sarcosine treatment **(E)**. The densitometry analysis of the blot (normalized to β-actin) verifies the enhanced activity of pmTOR **(F)**, pERK **(G)**, and pAkt **(H)** in each group of the experiments. (*p < 0.05; **p < 0.01; ***p < 0.001 compared with saline-treated group as assessed by *t*-test, Values shown are mean ± SEM, *n* = 10 for FST; *n* = 8 for EPM; *n* = 4 for western blots analysis per group).

Compared with controls treated with saline, rats treated with sarcosine (560 mg/kg) exhibited a significantly decreased immobility time in the FST (*t* = 4.404, df = 18, *p* < 0.001 on computer scoring and *t* = 2.857, df = 18, *p* < 0.05 on manual scoring), with concomitant significant increases in climbing behavior when injected with sarcosine 30 min before the FST (*t* = -2.545, df = 18, *p* < 0.05) (Figures 2A,B). In addition, to examine whether sarcosine could increase spontaneous locomotor activity and yield a false-positive result in the FST, the total closed arm entries and distance moved in EPM were measured as an indicator of general activity. However, sarcosine did not increase locomotor activity (Figures 2C,D).

To determine whether the decreased immobility was accompanied by increased activated mTOR signaling, the phosphorylated and activated forms of mTOR and mTOR upstream regulator proteins were assessed after acute sarcosine treatment. After the treatment, rats showed significant increases in the immunoreactivity of pmTOR, pERK, and pAkt, as would be expected with antidepressant-like effects (Figure 2E). Total mTOR, ERK, and Akt levels remained unchanged *in vivo*. Statistical analysis also confirmed a significant increase in pmTOR activity in sarcosine-treated rats (*t* = -253.445, df = 6, *p* < 0.001, Figure 2F). A similar expression trend was consistently observed in upstream regulators such as pERK (*t* = -6.536, df = 6, *p* < 0.01, Figure 2G) and pAkt (*t* = -3.741, df = 6, *p* < 0.05, Figure 2H).

### Role of activated AMPAR and mTOR signaling in the antidepressant-like effects of sarcosine

**Figure 3:**
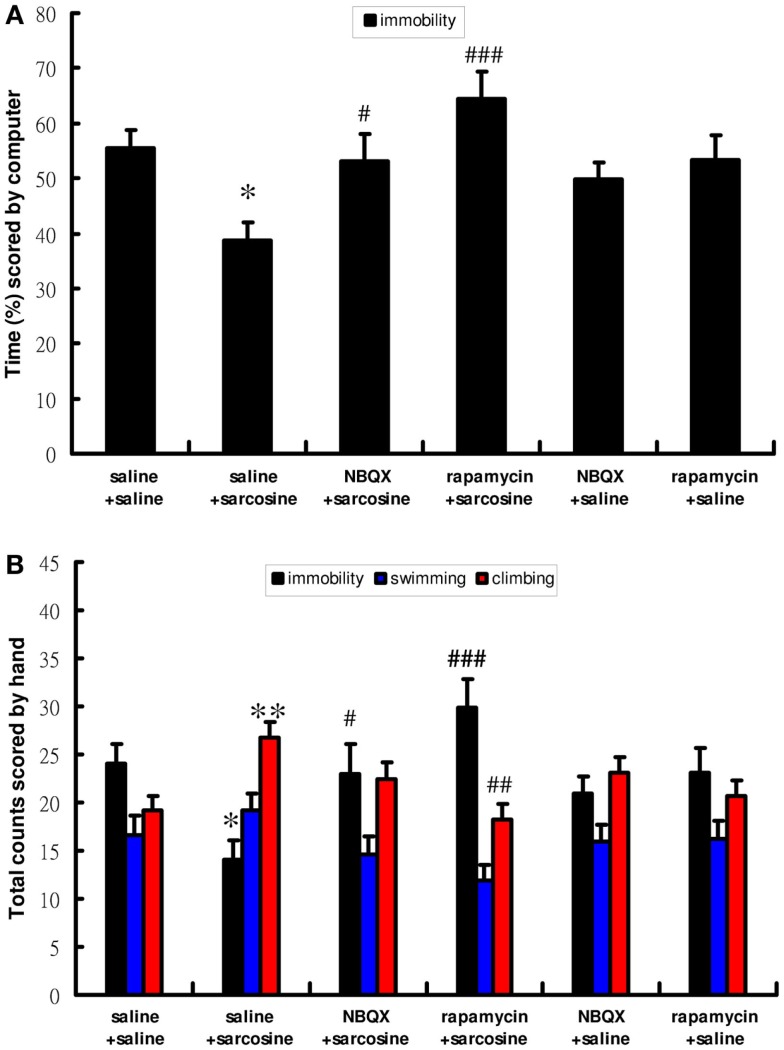
**The behaviors scored by computer (A) (percentage of immobility time) and manually (B) (frequency of immobility, swimming, climbing) of rats after acute sarcosine (560 mg/kg, i.p.) administration with pretreatment with NBQX (10 mg/kg, i.p.) or rapamycin (20 mg/kg, i.p.) in FST test**. Note that the decreased immobility and increased climbing resulted from acute sarcosine treatment is blocked when rats were pretreated with rapamycin. Similar effect is evidently observed when rats were pretreated with NBQX. (*p < 0.05; **p < 0.01 compared with saline/saline-treated group; ^#^*p* < 0.05, ^##^*p* < 0.01 compared with saline/sarcosine-treated group with Tukey *post hoc* analysis, Values shown are mean ± SEM, *n* = 8–9 per group).

To determine the role of mTOR signaling and AMPAR in antidepressant-like effects of sarcosine in the FST, rapamycin, an mTOR inhibitor, and NBQX, an AMPAR antagonist, were used. Similarly, acute sarcosine treatment significantly reduced immobility [computer scoring main effect: *F*(5,46) = 5.767; *p* < 0.001, Figure 3A; manual scoring main effect: *F*(5,46) = 5.545; *p* < 0.001, Figure 3B] and increased climbing behavior [manual scoring main effect: *F*(5,46) = 4.195; *p* < 0.01, Figure 3B]. Pretreatment with rapamycin (20 mg/kg, i.p.) completely blocked the sarcosine-elicited antidepressant-like effects of decreased immobility and increased climbing behavior in the FST (*p* > 0.05 on both computer and manual scorings for immobility; *p* > 0.05 on manual scoring for climbing behavior between rapamycin/sarcosine- and saline/saline-treated groups in Tukey *post hoc* analysis) (Figures 3A,B). In addition, a significant difference was observed between rapamycin/sarcosine- and saline/sarcosine-treated groups (*p* < 0.001 on computer and manual scorings for immobility; *p* < 0.01 on manual scoring for climbing behavior in Tukey *post hoc* analysis) (Figures 3A,B). Similarly, pretreatment with NBQX (10 mg/kg, i.p.) reversed decreased immobility and increased climbing behavior (*p* > 0.05 on both computer and manual scorings for immobility; *p* > 0.05 on manual scoring for climbing behavior between NBQX/sarcosine- and saline/saline-treated groups in Tukey *post hoc* analysis) (Figures 3A,B). In addition, a significant difference existed between NBQX/sarcosine- and saline/sarcosine-treated groups (*p* < 0.05 on both computer and manual scorings for immobility in Tukey *post hoc* analysis) (Figures 3A,B). These data indicated that mTOR signaling and AMPAR activations were required for the antidepressant-like effects of sarcosine in the FST.

### Role of AMPAR–mTOR signaling pathway in the antidepressant-like effects of sarcosine

**Figure 4:**
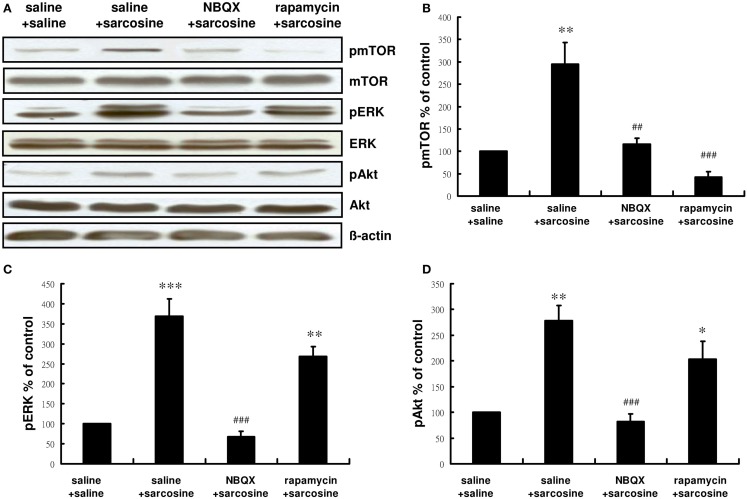
**Representative Western blotting (A) and relative expression ratio of pmTOR (B), pERK (C), and pAkt (D) in the hippocampus of rats after acute sarcosine (560 mg/kg, i.p.) administration with pretreatment with NBQX (10 mg/kg, i.p.) or rapamycin (20 mg/kg, i.p.)**. Western blots analysis shows a notably increased expression of pmTOR, pERK, and pAkt in rat hippocampus following acute sarcosine treatment. Note that the increased expression of pmTOR, pERK, and pAkt resulted from acute sarcosine treatment is blocked when rats were pretreated with NBQX. The increased expression of pmTOR resulted from acute sarcosine treatment is blocked when rats were pretreated with rapamycin and the increased expression of pERK and pAkt resulted from acute sarcosine treatment is not blocked (*p < 0.05; **p < 0.01 compared with saline/saline-treated group; ^##^*p* < 0.01, ^###^*p* < 0.001 compared with saline/sarcosine-treated group with Tukey *post hoc* analysis. Values shown are mean ± SEM, *n* = 4 per group).

We examined whether pretreatment with NBQX or rapamycin would affect the activity of the mTOR signaling pathway after sarcosine treatment. Similarly acute sarcosine treatment significantly increased immunoreactivity of pmTOR, pERK, and pAkt (Figure 4A). Total mTOR, ERK, and Akt levels remained unchanged *in vivo*. NBQX administration 30 min before sarcosine treatment blocked the sarcosine-elicited increase in the immunoreactivity of pmTOR [main effect: *F*(3,12) = 17.534, *p* < 0.001;*p* < 0.01 between NBQX/sarcosine- and saline/sarcosine-treated groups; *p* > 0.05 between NBQX/sarcosine- and saline/saline-treated groups in Tukey *post hoc* analysis; Figure 4B] as well as mTOR upstream regulator signaling kinases pERK [main effect: *F*(3,12) = 29.692, *p* < 0.001;*p* < 0.001 between NBQX/sarcosine- and saline/sarcosine-treated groups; *p* > 0.05 between NBQX/sarcosine- and saline/saline-treated groups in Tukey *post hoc* analysis; Figure 4C], and pAkt [main effect: *F*(3,12) = 14.969, *p* < 0.001;*p* < 0.001 between NBQX/sarcosine- and saline/sarcosine-treated groups; *p* > 0.05 between NBQX/sarcosine- and saline/saline-treated groups in Tukey *post hoc* analysis; Figure 4D] The increased immunoreactivity of pmTOR after sarcosine treatment was significantly abolished by pretreatment with rapamycin (*p* < 0.001 between rapamycin/saline- and saline/sarcosine-treated groups;*p* > 0.05 between rapamycin/saline- and saline/saline-treated groups in Tukey *post hoc* analysis; Figure 4B), but increased pERK and pAkt expression caused by acute sarcosine treatment was not blocked (both *p* > 0.05 between rapamycin/saline- and saline/sarcosine-treated groups in Tukey *post hoc* analysis; Figures 4C,D). These results suggested that sarcosine stimulated the mTOR signaling pathway that was directly dependent on AMPAR activation.

### Role of sarcosine treatment in GluR1 phosphorylation at the PKA site

**Figure 5:**
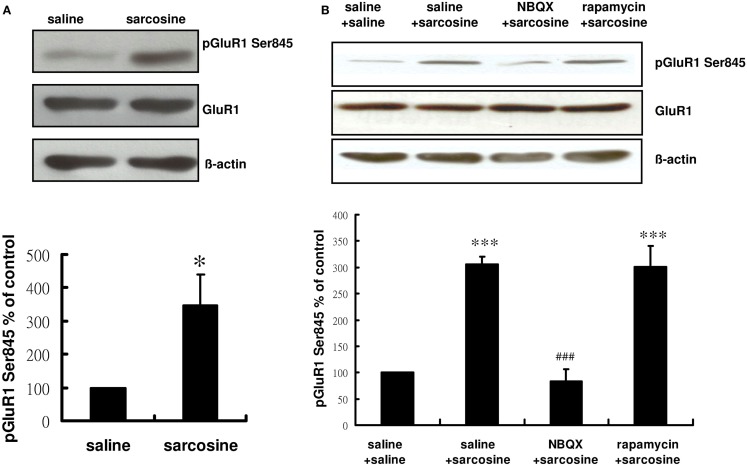
**Representative Western blotting of pGluR1 Ser845 from hippocampal slices of rats treated with saline or sarcosine (A) (560 mg/kg, i.p.) and after acute sarcosine (560 mg/kg, i.p.) administration with pretreatment with NBQX (10 mg/kg, i.p.) or rapamycin (20 mg/kg, i.p.) (B)**. Acute sarcosine treatment significantly increases the expression of pGluR1 Ser845. The increased expression of pGluR1 Ser845 resulted from acute sarcosine treatment is blocked when rats were pretreated with NBQX **(B)**. But, the effect was not blocked by pretreatment with rapamycin **(B)**. (*p < 0.05; **p < 0.01; ***p < 0.001 compared with saline/saline-treated group; ^###^*p* < 0.001 compared with saline/sarcosine-treated group with Tukey *post hoc* analysis. Values shown are mean ± SEM, *n* = 4 per group).

Glycine induces AMPAR membrane insertion *in vitro* (Lu et al., 2001), and the insertion of GluR1/GluR2-containing AMPAR in the neuronal membrane is controlled by the phosphorylation of the AMPAR subunit of GluR1 on its PKA site (GluR1 Ser845) (Banke et al., 2000; Esteban et al., 2003; Smith et al., 2006). Previous studies have shown that the activation of GluR1 Ser845 is correlated with the effects of several antidepressants (Du et al., 2006, 2007; Reus et al., 2011). Therefore, we determined whether sarcosine, a GlyT1 inhibitor (Smith et al., 1992) and NMDAR coagonist (Zhang et al., 2009), regulated the phosphorylation of GluR1 Ser845 after an acute *in vivo* sarcosine treatment of rats. Hippocampal phosphorylation states of GluR1 Ser845 were monitored in rats. The phosphorylation of hippocampal GluR1 Ser845 was significantly increased after an acute *in vivo* sarcosine treatment (*t* = -2.659, df = 6, *p* < 0.05; Figure 5A). NBQX administration 30 min before sarcosine treatment reversed the sarcosine-elicited increase in the immunoreactivity of pGluR1 Ser845 to the baseline. However, pretreatment with rapamycin did not block the sarcosine-elicited increase [main effect: *F*(3,12) = 26.324, *p* < 0.001;*p* > 0.05 between NBQX/sarcosine- and saline/saline-treated groups;*p* < 0.001 between NBQX/sarcosine- and saline/sarcosine-treated groups; *p* > 0.001 between rapamycin/sarcosine- and saline/saline-treated groups;*p* > 0.05 between rapamycin/sarcosine- and saline/sarcosine-treated groups in Tukey *post hoc* analysis; Figure 5B]. Total GluR1 levels remained unchanged *in vivo* (Figures 5A,B). These data indicated that sarcosine enhanced AMPAR membrane insertion via an AMPAR throughput.

## Discussion

We studied the effects of sarcosine, a GlyT1 inhibitor and NMDAR coagonist, at behavioral and molecular levels in rats. In addition, we demonstrated that a single injection of sarcosine exhibited antidepressant-like effects in an FST depression model and rapidly activated the mTOR signaling pathway. Sarcosine-induced antidepressant-like effects and increased mTOR signaling activation were significantly blocked by pretreatment with rapamycin or NBQX. In addition, pretreatment with NBQX also eliminated the ability of sarcosine to stimulate the activated form of mTOR as well as mTOR upstream regulator signaling kinases ERK and Akt. These results indicated that the antidepressant-like effects of sarcosine occurred through the activated AMPAR–mTOR signaling pathway. In addition, the phosphorylation of hippocampal AMPAR subunit GluR1 at its PKA site (often considered an indicator for GluR1 membrane insertion in neurons) was significantly increased after acute *in vivo* sarcosine treatment and abolished by pretreatment with NBQX. These data also demonstrated that sarcosine can enhance AMPAR membrane insertion through an AMPAR throughput.

First, consistent with the results of our and previous studies of NMDAR enhancers (Depoortere et al., 2005; Malkesman et al., 2012; Huang et al., 2013), we observed that sarcosine exhibited acute antidepressant-like effects in the FST (Figures 2A,B). Next, we investigated changes in the activation of the mTOR signaling pathway after a single injection of sarcosine. We observed that sarcosine rapidly increased the phosphorylated and activated forms of mTOR as well as mTOR upstream regulator signaling kinases ERK and Akt (Figures 2E–H). The direct role of mTOR signaling in the antidepressant-like effects of sarcosine on rats in the FST model was further examined using pretreatment with rapamycin, which resulted in a complete blockade of the antidepressant-like effects of sarcosine in the FST depression model (Figures 3A,B). These results demonstrated that the activation of mTOR signaling is necessary for the antidepressant-like effects of sarcosine. In addition, pretreatment with NBQX abolished the antidepressant-like effects of sarcosine (Figures 3A,B), demonstrating a requirement for the stimulation of AMPAR activity. The results demonstrated that sarcosine exerted rapid antidepressant-like effects mediated by the rapid activation of the mTOR signaling pathway and AMPAR stimulation.

Mammalian target of rapamycin is a serine/threonine protein kinase involved in cell proliferation, mortality, survival, and protein synthesis (Hay and Sonenberg, 2004), and dysregulation of its signaling cascade has been hypothesized to be a common pathophysiological feature of neuropsychiatric disorders (Hoeffer and Klann, 2010a). Rapid activation of the mTOR signaling pathway resulting in the rapid elevation of synapse-associated proteins represents a mechanism for the rapid antidepressant effect of the NMDAR antagonist ketamine (Li et al., 2010). A similar phenomenon has been evidenced in recent studies by using rapamycin blockade of metabotropic glutamate receptor mGluR_2/3_ antagonist LY341495, mGluR_5_ antagonist MTEP, mGluR_7_ agonist AMN082, and muscarinic receptor antagonist scopolamine (Dwyer et al., 2012; Voleti et al., 2013; Koike and Chaki, 2014; Palucha-Poniewiera et al., 2014). A postmortem study also showed considerable deficits in mTOR signaling in the prefrontal cortex of subjects diagnosed with major depressive disorder (Jernigan et al., 2011). Accumulating evidence suggests that the activation of mTOR signaling may be a convergent change induced by antidepressant drugs. As expected with antidepressant-like effects, our data are in agreement with those of other studies providing evidence that sarcosine-induced antidepressant-like effects are mediated by the activation of the mTOR signaling pathway. Furthermore, we assessed the possible involvement of AMPAR in the sarcosine-elicited activation of the mTOR signaling pathway. Inhibiting the AMPAR antagonist NBQX blocked the sarcosine-mediated induction of phosphorylated mTOR as well as mTOR upstream regulator signaling kinases, ERK and Akt. Our findings clearly demonstrated that the activation of the mTOR signaling pathway through AMPAR is involved in the antidepressant-like effects of sarcosine and, essentially, show a link with the mechanisms underlying the antidepressant-like effects of ketamine (Li et al., 2010).

**Figure 6:**
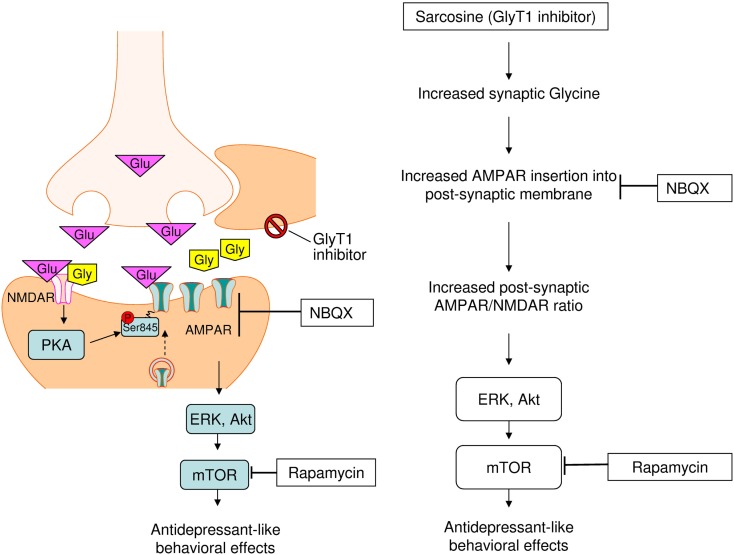
**Proposed cellular mechanisms underlying antidepressant-like effects of sarcosine**. Sarcosine caused the increased synaptic availability of glycine via inhibiting glycine uptake facilitates. The activation of synaptic NMDAR with co-agonist glycine induces AMPAR insertion into post-synaptic membrane that will amplify post-synaptic AMPAR levels, leading to increased AMPAR/NMDAR stimulation. Increased AMPAR throughput may ultimately cause downstream neuroproliferative effects through the activation of mTOR and multiple intracellular signaling cascades. Finally, sarcosine exhibit antidepressant-like property.

Similar to the NMDAR antagonist ketamine (Kugaya and Sanacora, 2005; Maeng et al., 2008; Li et al., 2010), the same antidepressant-like effects of sarcosine and other NMDAR enhancers have been previously noted at the behavioral level (Depoortere et al., 2005; Malkesman et al., 2012; Huang et al., 2013). We extended the same effect to molecular levels that demonstrated an increased sarcosine-elicited activation of the AMPAR–mTOR signaling pathway. The question raised is why the manipulation of the glutamatergic system by both NMDAR enhancers and blockades improves the symptoms of depression and shares final common targets AMPAR and mTOR for antidepressant effects. Preclinical evidence indicates that the mechanisms underlying the antidepressant effects of NMDAR antagonists are more complicated than those of a simple NMDAR blockade (Maeng and Zarate, 2007; Maeng et al., 2008). This finding suggests that the effects of ketamine are largely mediated through the interplay between AMPAR and NMDAR, and not through the NMDAR antagonist, thus finally inducing a rapid AMPAR-mediated synaptic potentiation. An *in vitro* study showed that AMPAR membrane insertion into the post-synaptic membrane can be induced by the activation of synaptic NMDAR with the coagonist glycine (Lu et al., 2001). The increased synaptic availability of glycine because of glycine uptake inhibition caused by sarcosine administration possibly facilitated AMPAR membrane insertion *in vivo*, as shown *in vitro*, and subsequently upregulated the receptors that amplify post-synaptic AMPAR levels, thus leading to increased AMPAR/NMDAR stimulation, previously hypothesized as a convergent mechanism for antidepressant effects (Zarate et al., 2006a; Schloesser et al., 2008). Increased AMPAR throughput may ultimately cause downstream neuroproliferative effects through the activation of mTOR and multiple intracellular signaling cascades. Therefore, sarcosine exhibits antidepressant-like effects as shown in Figure 6.

We examined the activation of GluR1 at the PKA site, an indicator for GluR1 membrane insertion in neurons, for assessing AMPAR trafficking into synapses after *in vivo* sarcosine treatment. Our data indicated that acute *in vivo* sarcosine treatment significantly increased the phosphorylation of GluR1 at the PKA site, reflecting enhanced AMPAR insertion into the membrane (Banke et al., 2000; Esteban et al., 2003; Smith et al., 2006). In addition, the sarcosine-elicited increased phosphorylation of GluR1 at the PKA site was blocked by NBQX pretreatment. These data suggested that sarcosine enhanced AMPAR membrane insertion through an AMPAR throughput. The results of this *in vivo* study along with those of *in vitro* studies of glycine-induced AMPAR insertion (Esteban et al., 2003; Smith et al., 2006) constitute crucial evidence explaining why sarcosine, through an NMDAR blockade, can exert antidepressant-like effects through the same AMPAR–mTOR signaling pathway.

Although cellular and behavioral studies of synaptic plasticity have demonstrated that the stimulation of mTOR signaling and synaptic protein synthesis is dependent on AMPAR activation (Hoeffer and Klann, 2010b; Livingstone et al., 2010), previous *in vitro* studies have demonstrated that mTOR predominantly regulates AMPAR trafficking (Wang et al., 2006), and mTOR activation increases the synaptic expression of various AMPAR subunits (Wang et al., 2006), including GluA1/GluA2 (Peng et al., 2011), GluR1 (Chen and Napoli, 2008; Slipczuk et al., 2009), and GluR2/3 (Wang et al., 2006). These results highlight a possible bidirectional regulation of the AMPAR–mTOR signaling pathway. However, our results showed that NBQX blocked the sarcosine-elicited increase in the immunoreactivity of pGluR1 Ser845 (Figure 5) and pmTOR (Figure 4B) as well as mTOR upstream regulator signaling kinases pERK (Figure 4C) and pAkt (Figure 4D). However, rapamycin could only block the increased immunoreactivity of pmTOR (Figure 4B). Rapamycin treatment did not significantly alter pGluR1 Ser845 levels (Figure 5) in sarcosine-treated rats. This *in vivo* study showed that sarcosine-induced AMPAR membrane insertion and mTOR activation required AMPAR activation; however, mTOR activation caused by sarcosine did not alter AMPAR membrane insertion.

Although there are potential parallels between the antidepressant mechanisms of sarcosine and ketamine, there are differences in the time scales in improving the depressive symptoms. Ketamine elicits an almost immediate antidepressant effect (Zarate et al., 2006b). We previously showed that although sarcosine exhibited faster therapeutic efficacy than an SSRI did, a longer time for onset of antidepressant effects (2–6 weeks) was observed with sarcosine treatment (Huang et al., 2013). Lamotrigine and riluzole, which take several weeks to exert antidepressant effects, show the same effects on AMPAR expression *in vivo* as sarcosine does (Du et al., 2007). Therefore, although a rapid increase in AMPAR:NMDAR ratio may be a common cellular mechanism of the antidepressant-like effect shared by sarcosine, ketamine, lamotrigine, and riluzole and a rapidly activated mTOR signaling pathway induced by a single dose of sarcosine in preclinical studies, only ketamine shows rapid onset of antidepressant action in clinical studies. However, whether ketamine’s clinical rapidity of action is attributable to a unique pharmacological characteristic, and not only to the rapidly activated AMPAR–mTOR signaling pathway showed in preclinical study, remains unknown; therefore, more clinical studies are needed to elucidate the neurobiological mechanisms underlying the different onset of antidepressant action.

Although the activation of the AMPAR–mTOR signaling pathway causes beneficial outcomes in the antidepressant-like effects of sarcosine, enhanced mTOR signaling may also cause side effects. The upregulated mTOR may accelerate tumor growth (Shor et al., 2009). Sarcosine activated prostate cancer cells (Dahl et al., 2011). Moreover, compared with benign prostate epithelial cells, sarcosine levels were increased in invasive prostate cancer cells (Sreekumar et al., 2009). Thus, there are safety clinical concerns regarding the use of antidepressants with a potential to enhance the mTOR signaling pathway, and further research is required.

We demonstrated, for the first time, that the activation of the AMPAR–mTOR signaling pathway after a single injection of sarcosine is required to produce antidepressant-like effects in the FST. In addition, sarcosine increased AMPAR membrane insertion, thus supporting our hypothesis that sarcosine triggers AMPAR membrane insertion, increases the AMPAR:NMDAR ratio, and activates the mTOR signaling pathway. However, our results would be more compelling if an entire series of studies of depression-related behaviors was conducted. In addition, the effects of sarcosine at behavioral and molecular levels were the same as those of ketamine. However, sarcosine and ketamine may have differential neurobiological effects in depression, and further research on this discrepancy is required. Nevertheless, the present evidence offers new insight into the antidepressant effect of glutamatergic system modulators, which may indicate an alternative glutamate-based approach for developing next-generation antidepressants.

## Author Contributions

Author K-TC, C-CH, and I-HW designed the study, wrote the protocol, and performed all the experiments and the statistical analysis. Author M-HT, M-JJ, and C-HW managed the literature searches and analyses. All authors have approved the final manuscript.

## Conflict of Interest Statement

The authors declare that the research was conducted in the absence of any commercial or financial relationships that could be construed as a potential conflict of interest.

## References

[B1] Banke T. G. Bowie D. Lee H. Huganir R. L. Schousboe A. Traynelis S. F. (2000). Control of GluR1 AMPA receptor function by cAMP-dependent protein kinase. J. Neurosci. 20, 89–102. 10627585 10.1523/JNEUROSCI.20-01-00089.2000 PMC6774102

[B2] Campbell S. Macqueen G. (2004). The role of the hippocampus in the pathophysiology of major depression. J. Psychiatry Neurosci. 29, 417–426. 15644983 PMC524959

[B3] Chen N. Napoli J. L. (2008). All-trans-retinoic acid stimulates translation and induces spine formation in hippocampal neurons through a membrane-associated RARalpha. FASEB J. 22, 236–245. 10.1096/fj.07-8739com 17712061

[B4] Cleary C. Linde J. A. Hiscock K. M. Hadas I. Belmaker R. H. Agam G. (2008). Antidepressive-like effects of rapamycin in animal models: implications for mTOR inhibition as a new target for treatment of affective disorders. Brain Res. Bull. 76, 469–473. 10.1016/j.brainresbull.2008.03.005 18534253

[B5] Cryan J. F. Page M. E. Lucki I. (2005). Differential behavioral effects of the antidepressants reboxetine, fluoxetine, and moclobemide in a modified forced swim test following chronic treatment. Psychopharmacology (Berl.) 182, 335–344. 10.1007/s00213-005-0093-5 16001105

[B6] Dahl M. Bouchelouche P. Kramer-Marek G. Capala J. Nordling J. Bouchelouche K. (2011). Sarcosine induces increase in HER2/neu expression in androgen-dependent prostate cancer cells. Mol. Biol. Rep. 38, 4237–4243. 10.1007/s11033-010-0442-2 21755295 PMC3402037

[B7] Depoortere R. Dargazanli G. Estenne-Bouhtou G. Coste A. Lanneau C. Desvignes C. (2005). Neurochemical, electrophysiological and pharmacological profiles of the selective inhibitor of the glycine transporter-1 SSR504734, a potential new type of antipsychotic. Neuropsychopharmacology 30, 1963–1985. 10.1038/sj.npp.1300772 15956994

[B8] Detke M. J. Johnson J. Lucki I. (1997). Acute and chronic antidepressant drug treatment in the rat forced swimming test model of depression. Exp. Clin. Psychopharmacol. 5, 107–112. 10.1037/1064-1297.5.2.107 9234045

[B9] Du J. Machado-Vieira R. Maeng S. Martinowich K. Manji H. K. Zarate C. A. Jr. (2006). Enhancing AMPA to NMDA throughput as a convergent mechanism for antidepressant action. Drug Discov. Today Ther. Strateg. 3, 519–526. 10.1016/j.ddstr.2006.11.012 25411578 PMC4234070

[B10] Du J. Suzuki K. Wei Y. Wang Y. Blumenthal R. Chen Z. (2007). The anticonvulsants lamotrigine, riluzole, and valproate differentially regulate AMPA receptor membrane localization: relationship to clinical effects in mood disorders. Neuropsychopharmacology 32, 793–802. 10.1038/sj.npp.1301178 16936714

[B11] Dwyer J. M. Lepack A. E. Duman R. S. (2012). mTOR activation is required for the antidepressant effects of mGluR(2)/(3) blockade. Int. J. Neuropsychopharmacol. 15, 429–434. 10.1017/s1461145711001702 22114864 PMC3580765

[B12] Encinas J. M. Fernandez A. P. Salas E. Castro-Blanco S. Munoz P. Rodrigo J. (2004). Nitric oxide synthase and NADPH-diaphorase after acute hypobaric hypoxia in the rat caudate putamen. Exp. Neurol. 186, 33–45. 10.1016/j.expneurol.2003.09.024 14980808

[B13] Esteban J. A. Shi S. H. Wilson C. Nuriya M. Huganir R. L. Malinow R. (2003). PKA phosphorylation of AMPA receptor subunits controls synaptic trafficking underlying plasticity. Nat. Neurosci. 6, 136–143. 10.1038/nn997 12536214

[B14] Fekadu A. Wooderson S. C. Markopoulo K. Donaldson C. Papadopoulos A. Cleare A. J. (2009). What happens to patients with treatment-resistant depression? A systematic review of medium to long term outcome studies. J. Affect. Disord. 116, 4–11. 10.1016/j.jad.2008.10.014 19007996

[B15] Hashimoto K. (2011). The role of glutamate on the action of antidepressants. Prog. Neuropsychopharmacol. Biol. Psychiatry 35, 1558–1568. 10.1016/j.pnpbp.2010.06.013 20600468

[B16] Hay N. Sonenberg N. (2004). Upstream and downstream of mTOR. Genes Dev. 18, 1926–1945. 10.1101/gad.1212704 15314020

[B17] Hoeffer C. A. Klann E. (2010a). mTOR signaling: at the crossroads of plasticity, memory and disease. Trends Neurosci. 33, 67–75. 10.1016/j.tins.2009.11.003 19963289 PMC2821969

[B18] Hoeffer C. A. Klann E. (2010b). mTOR signaling: at the crossroads of plasticity, memory, and disease. Trends Neurosci. 33, 67. 10.1016/j.tins.2009.11.003 19963289 PMC2821969

[B19] Hogg S. (1996). A review of the validity and variability of the elevated plus-maze as an animal model of anxiety. Pharmacol. Biochem. Behav. 54, 21–30. 10.1016/0091-3057(95)02126-4 8728535

[B20] Huang C. C. Wei I. H. Huang C. L. Chen K. T. Tsai M. H. Tsai P. (2013). Inhibition of glycine transporter-I as a novel mechanism for the treatment of depression. Biol. Psychiatry 74, 734–741. 10.1016/j.biopsych.2013.02.020 23562005

[B21] Jernigan C. S. Goswami D. B. Austin M. C. Iyo A. H. Chandran A. Stockmeier C. A. (2011). The mTOR signaling pathway in the prefrontal cortex is compromised in major depressive disorder. Prog. Neuropsychopharmacol. Biol. Psychiatry 35, 1774–1779. 10.1016/j.pnpbp.2011.05.010 21635931 PMC3154612

[B22] Koike H. Chaki S. (2014). Requirement of AMPA receptor stimulation for the sustained antidepressant activity of ketamine and LY341495 during the forced swim test in rats. Behav. Brain Res. 271, 111–115. 10.1016/j.bbr.2014.05.065 24909673

[B23] Krystal J. H. Sanacora G. Blumberg H. Anand A. Charney D. S. Marek G. (2002). Glutamate and GABA systems as targets for novel antidepressant and mood-stabilizing treatments. Mol. Psychiatry 7(Suppl. 1), S71–S80. 10.1038/sj.mp.4001021 11986998

[B24] Kugaya A. Sanacora G. (2005). Beyond monoamines: glutamatergic function in mood disorders. CNS Spectr. 10, 808–819. 16400244 10.1017/s1092852900010403

[B25] Li N. Lee B. Liu R. J. Banasr M. Dwyer J. M. Iwata M. (2010). mTOR-dependent synapse formation underlies the rapid antidepressant effects of NMDA antagonists. Science 329, 959–964. 10.1126/science.1190287 20724638 PMC3116441

[B26] Livingstone M. Atas E. Meller A. Sonenberg N. (2010). Mechanisms governing the control of mRNA translation. Phys. Biol. 7, 021001. 10.1088/1478-3975/7/2/021001 20463379

[B27] Lu W. Man H. Ju W. Trimble W. S. Macdonald J. F. Wang Y. T. (2001). Activation of synaptic NMDA receptors induces membrane insertion of new AMPA receptors and LTP in cultured hippocampal neurons. Neuron 29, 243–254. 10.1016/S0896-6273(01)00194-5 11182095

[B28] Maeng S. Zarate C. A. Jr. (2007). The role of glutamate in mood disorders: results from the ketamine in major depression study and the presumed cellular mechanism underlying its antidepressant effects. Curr. Psychiatry Rep. 9, 467–474. 10.1007/s11920-007-0063-1 18221626

[B29] Maeng S. Zarate C. A. Jr. Du J. Schloesser R. J. McCammon J. Chen G. (2008). Cellular mechanisms underlying the antidepressant effects of ketamine: role of alpha-amino-3-hydroxy-5-methylisoxazole-4-propionic acid receptors. Biol. Psychiatry 63, 349–352. 10.1016/j.biopsych.2007.05.028 17643398

[B30] Malkesman O. Austin D. R. Tragon T. Wang G. Rompala G. Hamidi A. B. (2012). Acute d-serine treatment produces antidepressant-like effects in rodents. Int. J. Neuropsychopharmacol. 15, 1135–1148. 10.1017/s1461145711001386 21906419 PMC3278496

[B31] Nestler E. J. Barrot M. Dileone R. J. Eisch A. J. Gold S. J. Monteggia L. M. (2002). Neurobiology of depression. Neuron 34, 13–25. 10.1016/S0896-6273(02)00653-0 11931738

[B32] Palucha-Poniewiera A. Szewczyk B. Pilc A. (2014). Activation of the mTOR signaling pathway in the antidepressant-like activity of the mGlu5 antagonist MTEP and the mGlu7 agonist AMN082 in the FST in rats. Neuropharmacology 82, 59–68. 10.1016/j.neuropharm.2014.03.001 24631968

[B33] Peng X. Kim J. Zhou Z. Fink D. J. Mata M. (2011). Neuronal Nogo-A regulates glutamate receptor subunit expression in hippocampal neurons. J. Neurochem. 119, 1183–1193. 10.1111/j.1471-4159.2011.07520.x 21985178 PMC3235679

[B34] Porsolt R. D. Bertin A. Jalfre M. (1977). Behavioral despair in mice: a primary screening test for antidepressants. Arch. Int. Pharmacodyn. Ther. 229, 327–336. 596982

[B35] Reus G. Z. Stringari R. B. Ribeiro K. F. Ferraro A. K. Vitto M. F. Cesconetto P. (2011). Ketamine plus imipramine treatment induces antidepressant-like behavior and increases CREB and BDNF protein levels and PKA and PKC phosphorylation in rat brain. Behav. Brain Res. 221, 166–171. 10.1016/j.bbr.2011.02.024 21397634

[B36] Rizvi S. J. Grima E. Tan M. Rotzinger S. Lin P. McIntyre R. S. (2014). Treatment-resistant depression in primary care across Canada. Can. J. Psychiatry 59, 349–357. 25007419 10.1177/070674371405900702 PMC4086317

[B37] Rodgers R. J. Johnson N. J. (1995). Factor analysis of spatiotemporal and ethological measures in the murine elevated plus-maze test of anxiety. Pharmacol. Biochem. Behav. 52, 297–303. 10.1016/0091-3057(95)00138-M 8577794

[B38] Schloesser R. J. Huang J. Klein P. S. Manji H. K. (2008). Cellular plasticity cascades in the pathophysiology and treatment of bipolar disorder. Neuropsychopharmacology 33, 110–133. 10.1038/sj.npp.1301575 17912251

[B39] Shimizu-Sasamata M. Kawasaki-Yatsugi S. Okada M. Sakamoto S. Yatsugi S. Togami J. (1996). YM90K: pharmacological characterization as a selective and potent alpha-amino-3-hydroxy-5-methylisoxazole-4-propionate/kainate receptor antagonist. J. Pharmacol. Exp. Ther. 276, 84–92. 8558460

[B40] Shor B. Gibbons J. J. Abraham R. T. Yu K. (2009). Targeting mTOR globally in cancer: thinking beyond rapamycin. Cell Cycle 8, 3831–3837. 10.4161/cc.8.23.10070 19901542

[B41] Skolnick P. (1999). Antidepressants for the new millennium. Eur. J. Pharmacol. 375, 31–40. 10.1016/S0014-2999(99)00330-1 10443562

[B42] Slipczuk L. Bekinschtein P. Katche C. Cammarota M. Izquierdo I. Medina J. H. (2009). BDNF activates mTOR to regulate GluR1 expression required for memory formation. PLoS ONE 4:e6007. 10.1371/journal.pone.0006007 19547753 PMC2695538

[B43] Smith K. E. Borden L. A. Hartig P. R. Branchek T. Weinshank R. L. (1992). Cloning and expression of a glycine transporter reveal colocalization with NMDA receptors. Neuron 8, 927–935. 10.1016/0896-6273(92)90207-T 1534013

[B44] Smith K. E. Gibson E. S. Dell’acqua M. L. (2006). cAMP-dependent protein kinase postsynaptic localization regulated by NMDA receptor activation through translocation of an A-kinase anchoring protein scaffold protein. J. Neurosci. 26, 2391–2402. 10.1523/jneurosci.3092-05.2006 16510716 PMC6793655

[B45] Sreekumar A. Poisson L. M. Rajendiran T. M. Khan A. P. Cao Q. Yu J. (2009). Metabolomic profiles delineate potential role for sarcosine in prostate cancer progression. Nature 457, 910–914. 10.1038/nature07762 19212411 PMC2724746

[B46] Stewart C. A. Reid I. C. (2002). Antidepressant mechanisms: functional and molecular correlates of excitatory amino acid neurotransmission. Mol. Psychiatry 7(Suppl. 1), S15–S22. 10.1038/sj.mp.4001014 11986991

[B47] Tokita K. Yamaji T. Hashimoto K. (2012). Roles of glutamate signaling in preclinical and/or mechanistic models of depression. Pharmacol. Biochem. Behav. 100, 688–704. 10.1016/j.pbb.2011.04.016 21536063

[B48] Trivedi M. H. Fava M. Wisniewski S. R. Thase M. E. Quitkin F. Warden D. (2006). Medication augmentation after the failure of SSRIs for depression. N. Engl. J. Med. 354, 1243–1252. 10.1056/NEJMoa052964 16554526

[B49] Voleti B. Navarria A. Liu R. J. Banasr M. Li N. Terwilliger R. (2013). Scopolamine rapidly increases mammalian target of rapamycin complex 1 signaling, synaptogenesis, and antidepressant behavioral responses. Biol. Psychiatry 74, 742–749. 10.1016/j.biopsych.2013.04.025 23751205 PMC3773272

[B50] Wang Y. Barbaro M. F. Baraban S. C. (2006). A role for the mTOR pathway in surface expression of AMPA receptors. Neurosci. Lett. 401, 35–39. 10.1016/j.neulet.2006.03.011 16677760

[B51] Zarate C. A. Jr. Singh J. Manji H. K. (2006a). Cellular plasticity cascades: targets for the development of novel therapeutics for bipolar disorder. Biol. Psychiatry 59, 1006–1020. 10.1016/j.biopsych.2005.10.021 16487491

[B52] Zarate C. A. Jr. Singh J. B. Carlson P. J. Brutsche N. E. Ameli R. Luckenbaugh D. A. (2006b). A randomized trial of an N-methyl-d-aspartate antagonist in treatment-resistant major depression. Arch. Gen. Psychiatry 63, 856–864. 10.1001/archpsyc.63.8.856 16894061

[B53] Zarate C. A. Jr. Singh J. B. Quiroz J. A. De Jesus G. Denicoff K. K. Luckenbaugh D. A. (2006c). A double-blind, placebo-controlled study of memantine in the treatment of major depression. Am. J. Psychiatry 163, 153–155. 10.1176/appi.ajp.163.1.153 16390905

[B54] Zhang H. X. Hyrc K. Thio L. L. (2009). The glycine transport inhibitor sarcosine is an NMDA receptor co-agonist that differs from glycine. J. Physiol. 587, 3207–3220. 10.1113/jphysiol.2009.168757 19433577 PMC2727032

